# Definitive management of gallstone pancreatitis in England

**DOI:** 10.1308/003588412X13171221591934

**Published:** 2012-09

**Authors:** Y El-Dhuwaib, M Deakin, GG David, D Durkin, DJ Corless, JP Slavin

**Affiliations:** ^1^Mid Cheshire Hospitals NHS Foundation Trust,UK; ^2^University Hospital of North Staffordshire NHS Trust,UK

**Keywords:** Gallstones, Pancreatitis, Cholecystectomy, Endoscopic sphincterotomy

## Abstract

**INTRODUCTION:**

The aim of this study was to investigate whether definitive treatment of gallstone pancreatitis (GSP) by either cholecystectomy or endoscopic sphincterotomy in England conforms with British Society of Gastroenterology (BSG) guidelines and to validate these guidelines.

**METHODS:**

Hospital Episode Statistics data were used to identify patients admitted for the first time with GSP between April 2007 and April 2008. These patients were followed until April 2009 to identify any who underwent definitive treatment or were readmitted with a further bout of GSP as an emergency.

**RESULTS:**

A total of 5,454 patients were admitted with GSP between April 2007 and April 2008, of whom 1,866 (34.2%) underwent definitive treatment according to BSG guidelines, 1,471 on the index admission. Patients who underwent a cholecystectomy during the index admission were less likely to be readmitted with a further bout of GSP (1.7%) than those who underwent endoscopic sphincterotomy alone (5.3%) or those who did not undergo any form of definitive treatment (13.2%). Of those patients who did not undergo definitive treatment before discharge, 2,239 received definitive treatment following discharge but only 395 (17.6%) of these had this within 2 weeks. Of the 505 patients who did not undergo definitive treatment on the index admission and who were readmitted as an emergency with GSP, 154 (30.5%) were admitted during the 2 weeks immediately following discharge.

**CONCLUSIONS:**

Following an attack of mild GSP, cholecystectomy should be offered to all patients prior to discharge. If patients are not fit for surgery, an endoscopic sphincterotomy should be performed as definitive treatment.

Acute pancreatitis is associated with considerable morbidity and mortality.^1–3^ Gallstones are the aetiological factor in 30–50% of cases.^2,4,5^ Stones less than 5mm in diameter, a wide cystic duct and a longer common channel between the bile and pancreatic duct are predisposing factors.^6^

UK guidelines for the management of gallstone pancreatitis (GSP) were first published by the British Society of Gastroenterology (BSG) in 1998^7^ and then amended in 2005.^8^ These guidelines suggest that all patients with mild GSP should be offered definitive treatment: cholecystectomy if they are fit for surgery or endoscopic sphincterotomy (ES) if not. Definitive treatment should be performed during the index admission or within two weeks of discharge. Following severe GSP, the guidelines suggest cholecystectomy should be delayed until the patient is fully recovered. However, patients with predicted severe GSP or with cholangitis should have an early ES as part of their initial management.^9–11^

Published studies suggest that adherence to the BSG guidelines in the UK is variable; definitive treatment performed in accordance with the guidelines varies from 6.6% to 89%^12–15^ while in the US it is 50%.^16^ There are no national data available on the definitive treatment of gallstones following an attack of GSP or on readmission/mortality rates among patients in whom there was a delay in management.

The appropriate timing of definitive treatment is not yet established. One study found that 31% of recurrent GSP occurred in the first two weeks following discharge.^17^ In another study when patients were discharged home but operated on within two weeks this figure was 6.5%.^18^ This further study also suggested that performing definitive treatment during the index admission increases the length of hospital stay (LOS).

Our study investigated current practice with regard to definitive treatment of GSP in England with reference to BSG guidelines.^8^ It also investigated the effectiveness of cholecystectomy and ES in preventing a further attack of GSP and the consequences of delayed treatment.

## Methods

Hospital Episode Statistics (HES) data for the financial year April 2007 to March 2008 were imported into Microsoft SQL server for analysis. HES contains information on all patients treated in England in National Health Service (NHS) hospitals and those NHS patients treated in the private sector. Patients admitted with gallstones and acute pancreatitis as an emergency were identified by searching the admission method, diagnostic and operative fields.

To identify an emergency admission the method of admission was searched for codes 21, 22, 23, 24 and 28. The International Classification of Diseases (ICD-10) codes K85* and K80* were used to identify acute pancreatitis and cholelithiasis respectively. Operative procedures were identified using the Office of Population Censuses and Surveys (OPCS-4) codes: J18* was used to identify cholecystectomy and J38* to identify ES. Individual patients were followed across time and place using ‘HESID’, a unique number generated by combination of the patient’s NHS number, local patient identifier, postcode, sex and date of birth.

The index cohort consisted of patients admitted as an emergency for the first time with GSP between April 2007 and April 2008. Any patients admitted with GSP or who had an intervention (ES or cholecystectomy) between April 2005 and April 2007 were excluded from this cohort. The cohort was followed until April 2009 (median duration: 18 months, range: 12–24 months) to identify those who underwent cholecystectomy or ES or those who were readmitted with GSP as an emergency.

The time to definitive treatment was defined as the time from discharge until ES or cholecystectomy was performed. If a patient underwent ES and cholecystectomy, the date of the first treatment was used. If a patient was readmitted with a further bout of GSP as an emergency, the time from initial discharge until the first emergency readmission was used as the time until the second attack. Patients were stratified into four groups based on LOS. All statistical analyses were performed using SPSS® version 18.0 (SPSS, Chicago, IL, US).

## Results

**Figure 1:**
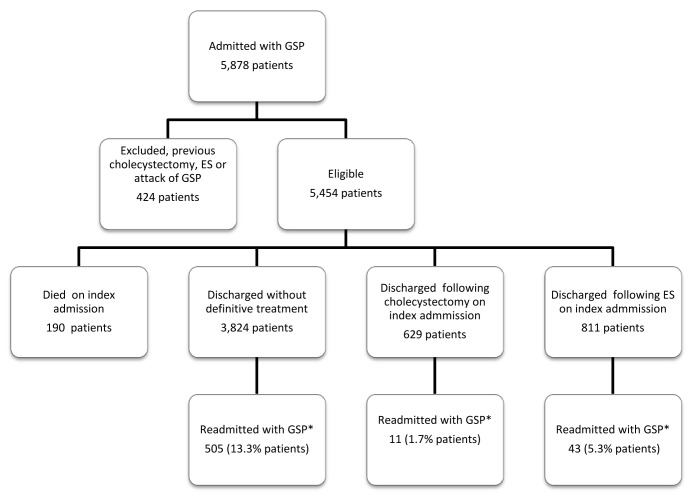
Study flowchart

A total of 5,878 patients were admitted as an emergency with acute GSP between April 2007 and April 2008. After excluding patients who had been admitted with GSP or who had undergone a cholecystectomy/ES during the period April 2005 to April 2007, 5,454 patients remained for the final analysis (Fig 1). The median age of the cohort was 63 years. Women were nearly twice as likely to be admitted with GSP as men and the median LOS was one week. During the index admission, 190 patients (3.5%) died. Of these, 25 had already undergone an ES, 6 a cholecystectomy and 159 no definitive treatment. The median number of patients admitted with GSP to each NHS trust was 35 patients per year (interquartile range [IQR]: 25–51) (Table 1).

**Table 1:** Demographics of patients admitted with gallstone pancreatitis (GSP) between April 2007 and April 2008 and followed until April 2009

Number of patients	5,454
Median age (IQR)	63 years (45–76 years)
Male-to-female ratio	1:1.73
Median length of stay (IQR)	7 days (4–12 days)
In-hospital mortality	190 patients (3.5%)
Median number of admissions per trust (IQR)	34 (24–51)
Number of patients receiving definitive treatment on the index admission	1,471
Number of patients receiving definitive treatment during study period	4,105
Number of readmissions as an emergency with GSP	559
Number of deaths following readmission	22
Median number of readmissions with GSP (range)	1 (1–3)

IQR = interquartile range

The majority (4,105 patients) underwent definitive treatment either within BSG guidelines (*n*=1,866) or at a later date (*n*=2,239). Two-thirds (*n*=2,706, 65.9%) underwent a cholecystectomy while 713 patients (17.3%) had an ES and 686 (16.7%) underwent both procedures. Patients who underwent a cholecystectomy (median age: 56 years, IQR: 39–68 years) were significantly younger than those who underwent an ES alone (median age: 78 years, IQR: 69–84 years) and those who did not undergo definitive treatment (median age: 72 years, IQR 56–83 years). On the index admission, 1,471 patients underwent definitive treatment. Of those who were discharged, 811 underwent an ES alone and 629 a cholecystectomy (28 of these underwent both an ES and a cholecystectomy).

A total of 559 patients, 505 of whom had not undergone definitive treatment on the index admission, were readmitted 655 times with a further attack of GSP by April 2009 (median number of readmissions: 1, range: 1–3 readmissions) and 22 patients (3.9%) died following a readmission with GSP (Table 1). Patients who underwent a cholecystectomy during the index admission had a significantly lower readmission rate (1.7%) compared with those who underwent an ES alone (5.3%) and those who did not have any form of definitive treatment during the index admission (13.2%) (Fig 1).

**Figure 2:**
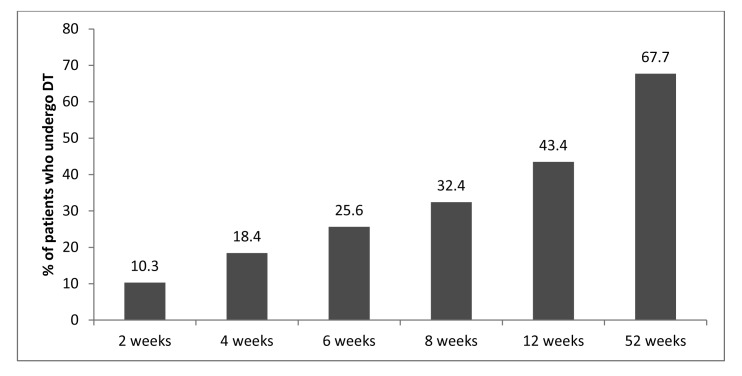
Cumulative percentage of patients who undergo definitive treatment in those who did not receive definitive treatment on the index admission

**Figure 3:**
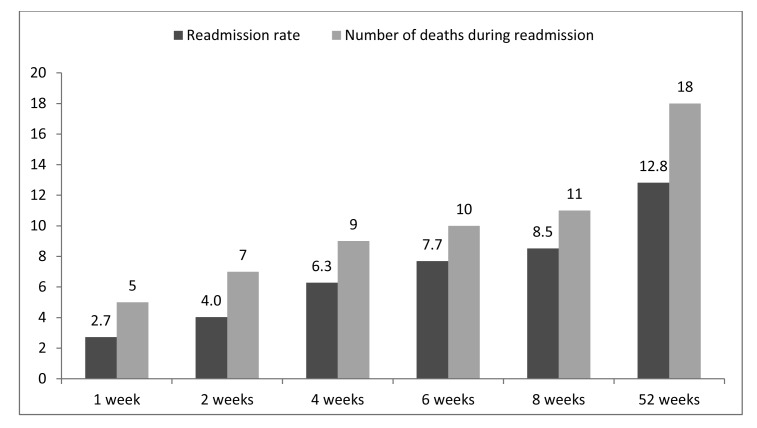
Cumulative readmission rate for gallstone pancreatitis and number of deaths in patients who did not receive definitive treatment on the index admission

Approximately a third of patients (*n*=1,866, 34.2%) underwent definitive treatment according to BSG guidelines (ie on the index admission or within two weeks of discharge). The majority of these patients (*n*=1,471) had definitive treatment during the index admission. Of the 3,824 patients discharged without definitive treatment, only 10.3% underwent definitive treatment within the next two weeks and only 32.4% had undergone definitive treatment by eight weeks (Fig 2). Of the 505 patients who did not undergo definitive treatment and who were readmitted with a further diagnosis of GSP, about a third of these (*n*=154, 30.5%) were readmitted during the first two weeks following discharge and seven died during this readmission. By eight weeks, the cumulative readmission rate in patients who did not undergo definitive treatment on the index admission was 8.5% (Fig 3).

**Figure 4:**
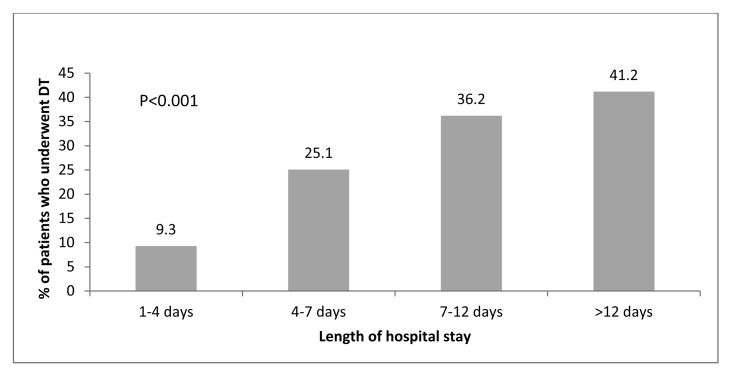
The proportion of patients who underwent definitive treatment during the index admission

In Figure 4 patients are stratified into four groups according to their LOS. Only 9.3% of those who stayed four days or less underwent definitive treatment on the index admission compared with 41.2% of those who stayed more than twelve days.

## Discussion

Data derived from HES are used increasingly to investigate delivery of care in England. The validity of studies using these data depends on the accuracy and depth of coding, and this has been questioned. Campbell *et al* showed in a systematic review that there is generally a high level of accuracy (91%) for diagnosis although the accuracy for coding of operations or procedures was only 69.5%.^19^ There may have been an improvement in the accuracy of coding in the NHS in England in the ten years since this study due to the introduction of the payment by results scheme, which relies on data derived from OPCS-4 codes.

Since 2007–2008, the Audit Commission has conducted an annual audit of clinical coding in England. Results from the first of these audits suggest that 10.5% of primary procedures are coded incorrectly although there was wide variation between different trusts and the inaccuracies did not necessarily mean that patients were categorised incorrectly.^20^ Another study looking at aortic aneurysm surgery found that coding accuracy appeared to be high if diagnostic, operative and administrative codes were compared, and accuracy could be improved further if they were combined,^21^ similar to our study.

Despite improvements, HES data need careful interpretation. Variations in coding are usually ignored when large aggregations of data are used, for example at national level as in this study. In this situation, the variations in coding are likely to occur randomly and therefore to cancel each other out. Conversely, if comparisons were made between individual providers, then variation in coding could not be ignored in this way.^22,23^ We have not attempted any such comparisons.

This national audit shows that current practice in England with regard to the definitive management of patients with GSP falls well short of that suggested by the BSG.^8^ In fact, only a third of patients received definitive treatment on the index admission or within two weeks of discharge.

HES data lack many clinical details that have been used in other comparative studies of acute pancreatitis to stratify patient populations into predicted severe and mild disease, and this is a limitation of uor study. The majority of patients admitted with GSP will, however, have mild disease and should be undergoing treatment in line with the BSG guidelines.

This study demonstrates that patients who had definitive treatment during the index admission are less likely to be readmitted with GSP than those who did not. When the issue of timing is addressed, the study also reveals that a third of readmissions with GSP occur in the two weeks following discharge and that some of these patients died. Furthermore, only 10% of the patients discharged following an index admission with GSP who did not undergo definitive treatment on this admission underwent definitive treatment in the two weeks following discharge (Fig 2). This suggests that clinicians are not making proper use of the facility provided by the BSG guidelines to undertake a cholecystectomy within two weeks of discharge on a routine list.

Although there was a general consensus among clinicians who prepared the BSG guidelines that definitive treatment was best performed during the index admission or within two weeks of discharge, this recommendation was based on expert opinion and not objective data. It may be that definitive treatment during the index admission is advisable and that patients suffering an attack of mild GSP should have a cholecystectomy or ES before discharge. Subsequent guidelines in acute pancreatitis should possibly take this into account.

In addition, it appears that once patients were discharged without definitive treatment, only a third had undergone definitive treatment within two months of discharge. This may reflect the lack of available operating time on routine lists together with poor prioritisation. On the other hand, this and other observational studies^18,24^ have shown that the LOS during the index admission increases if patients undergo definitive treatment during that admission. It may be necessary to book patients with mild GSP for a cholecystectomy once the diagnosis has been made, even if they are still settling, as this has been shown to be safe and reduce LOS.^25,26^ In severe pancreatitis early cholecystectomy should be avoided while the patient is recovering; there may, however, be a role for initial treatment of these patients with an ES to modify this attack and to prevent further attacks with interval cholecystectomy at a later date.^27–29^

There is still considerable debate as to whether an ES reduces the risk of a further bout of GSP to the same level as a cholecystectomy.^30–35^ This study has shown that cholecystectomy is superior to ES with regard to the prevention of further attacks of GSP. Furthermore, cholecystectomy is a lower risk procedure and the later biliary complications attributable to gallstones in the gallbladder such as cholecystitis are avoided.^24^ There is therefore general agreement that all patients with acute GSP who are fit enough should undergo a cholecystectomy. Delaying surgery will not save treatment costs although it may decrease LOS on the index admission. Delaying definitive treatment will, however, increase the possibility of a further emergency readmission with GSP with the associated costs, morbidity and mortality, and this will increase the burden on emergency services.

The median number of patients admitted as an emergency with GSP per NHS trust is 35 (IQR: 25–51). Therefore, for the majority of NHS trusts, the extra theatre time required is less than one operation per week, which in most cases should be managed easily if treatment of GSP were given the appropriate clinical priority.^36^

## Conclusions

Following an attack of mild GSP, cholecystectomy should be offered to all patients prior to discharge and this should be prioritised appropriately on emergency or elective lists. If a patient is not fit for surgery, an ES should be performed
